# Cytosine Methylation Dysregulation in Neonates Following Intrauterine Growth Restriction

**DOI:** 10.1371/journal.pone.0008887

**Published:** 2010-01-26

**Authors:** Francine Einstein, Reid F. Thompson, Tushar D. Bhagat, Melissa J. Fazzari, Amit Verma, Nir Barzilai, John M. Greally

**Affiliations:** 1 Department of Obstetrics, Gynecology and Women's Health, Albert Einstein College of Medicine, Bronx, New York, United States of America; 2 Department of Genetics (Computational Genetics), Albert Einstein College of Medicine, Bronx, New York, United States of America; 3 Department of Developmental and Molecular Biology, Albert Einstein College of Medicine, Bronx, New York, United States of America; 4 Department of Epidemiology and Population Health, Albert Einstein College of Medicine, Bronx, New York, United States of America; 5 Department of Medicine, and Center for Epigenomics, Albert Einstein College of Medicine, Bronx, New York, United States of America; Victor Chang Cardiac Research Institute, Australia

## Abstract

**Background:**

Perturbations of the intrauterine environment can affect fetal development during critical periods of plasticity, and can increase susceptibility to a number of age-related diseases (*e.g.,* type 2 diabetes mellitus; T2DM), manifesting as late as decades later. We hypothesized that this biological memory is mediated by permanent alterations of the epigenome in stem cell populations, and focused our studies specifically on DNA methylation in CD34+ hematopoietic stem and progenitor cells from cord blood from neonates with intrauterine growth restriction (IUGR) and control subjects.

**Methods and Findings:**

Our epigenomic assays utilized a two-stage design involving genome-wide discovery followed by quantitative, single-locus validation. We found that changes in cytosine methylation occur in response to IUGR of moderate degree and involving a restricted number of loci. We also identify specific loci that are targeted for dysregulation of DNA methylation, in particular the hepatocyte nuclear factor 4α (*HNF4A*) gene, a well-known diabetes candidate gene not previously associated with growth restriction *in utero*, and other loci encoding *HNF4A*-interacting proteins.

**Conclusions:**

Our results give insights into the potential contribution of epigenomic dysregulation in mediating the long-term consequences of IUGR, and demonstrate the value of this approach to studies of the fetal origin of adult disease.

## Introduction

The concept of fetal origin of adult disease suggests that early life conditions can “program” the fetus for a spectrum of adverse health outcomes as an adult [1]. The link between intrauterine growth restriction (IUGR) and adult diseases such as type 2 diabetes and cardiovascular disease has been extensively supported by epidemiological [2], [3], [4], [5], [6], [7] and animal [8], [9], [10], [11] studies. To date, reports have focused on characterizing the pathophysiological consequences of an adverse intrauterine environment, and have revealed dysregulation of gene expression and a variety of functional impairments in individual tissues (including liver [12], [13], skeletal muscle [4], [14], pancreas [15], [16], kidney [17], and bone [18]) that precede and are potentially contributory to a range of later adult diseases. Taken together, these studies define a precocious aging phenotype, culminating in an increased risk of premature death [10], [19].

Permanent and sometimes progressive changes in gene expression have been observed in multiple tissues as a consequence of IUGR [16], [17], [20], [21]. Dysregulation of the epigenome may explain changes that are propagated from parent to daughter cells in IUGR offspring throughout life. For instance, offspring of rats fed a restricted protein diet throughout pregnancy showed changes in DNA methylation at multiple genes, with corresponding changes in gene expression, both of which were prevented by maternal supplementation of folic acid (an important methyl donor), further implicating an epigenetic mechanism [22], [23], [24]. Additionally, human studies also demonstrated epigenetic differences in response to an adverse intrauterine environment, as periconceptional exposure to famine was associated with altered DNA methylation at multiple sites within the known *IGF2* differentially methylated region (DMR) [25]. Similar epigenetic dysregulation has been observed in a variety of tissues [23], [24], [26], [27], consistent with IUGR-induced susceptibility to age-related diseases affecting multiple organ systems.

As the life-long progenitors of many differentiated lineages, stem cells must act as custodians of epigenomic regulatory patterns in order that these changes persist in most tissues throughout the lifetime of an organism. Hematopoietic stem (CD34+) cells are one of the best-characterized stem cell types, and represent a neonatally accessible population, present as ∼1% of mononucleated umbilical cord blood cells [28]. Following tissue injury, this stem cell population is able to mobilize, proliferate, and home to target microenvironments at the site of damage [29], and may coordinate injury repair by stimulating tissue-specific progenitors [30], [31]. Moreover, these stem cells are multipotent progenitors of the immune system, which itself is likely to mediate inflammation, development, and progression of T2DM and cardiovascular disease [32].

To determine whether an adverse intrauterine environment leads to epigenetic changes that have functional pathological consequences, we studied DNA methylation in multipotent hematopoietic (CD34+) stem cells of IUGR neonates and matched controls. We employed our new, high-resolution version of the microarray-based HELP assay [33] to study ∼1.32 million loci throughout the human genome. Rather than focus on known candidate loci, we expanded the search genome-wide to identify novel loci involved and also tested for global, non-specific effects of IUGR on cytosine methylation. We followed this screening approach with a second stage of analysis, validating methylation status using quantitative, nucleotide-resolution bisulphite MassArray [34] to define the dysregulatory effects of IUGR on the epigenome.

## Methods

This study was approved by the institutional review board (IRB) of the Montefiore Medical Center and the Committee on Clinical Investigation at the Albert Einstein College of Medicine and is in accordance with Health Insurance Portability and Accountability Act (HIPAA) regulations. Written informed consent was obtained from all subjects prior to participation.

### Clinical Data Collection and Identification of Cases and Controls

Biological samples and clinical information were collected from consenting women who delivered IUGR or infants with appropriate growth (matched for gestational age at delivery, ethnicity and gender). Both birth weight and ponderal index (a measurement of neonatal weight relative to length) were used to identify cases and controls. IUGR had birth weight and ponderal index <10th percentile for gestational age and gender and controls had normal percentiles (>10th and <90th) for both parameters. Pertinent data on maternal medical history, pregnancy complications and neonatal hospital stay were recorded.

### Isolation of CD34+ Cells

CD34+ cells, which constitute approximately 1% of nucleated blood cells in umbilical cord blood [28], were isolated from the cord blood specimen using an immunomagnetic separation technique, as previously described [35]. Mononuclear cells are separated by Ficoll-Paque density gradient, following which CD34+ cells are obtained by positive immunomagnetic bead selection, using Macs columns (Miltenyi Biotech). The isolated cells having >95% purity [36], [37] are cryopreserved in 10% DMSO by controlled rate freezing.

### HELP Assay

High-resolution HELP assays were performed according to recent advances in the technology [33]. Genomic DNA was isolated, digested to completion by either HpaII or MspI separately, and then ligated to a mixture of two oligonucleotide pair adapters, each complementary to the cohesive ends generated by the restriction digest. The adapters then served as a priming site for a PCR reaction (ligation-mediated PCR, LM-PCR) that we have described to generate a product predominantly in the 50–2,000 bp size range [33]. Following PCR, the HpaII and MspI representations were labeled with different fluorophores using random priming and were then cohybridized on a customized genomic microarray representing ∼1.32 million HpaII/MspI fragments of 50–2,000 bp in unique sequence [33].

### Data Analysis

Microarray data were pre-processed and subject to quality control and quantile normalization as previously described [38]. HpaII/MspI ratio values were compared between groups for all loci throughout the genome using a paired t-test with cases (n = 5) and controls (n = 5) matched by gender and gestational age, as well as ethnicity where possible (see **Table S1**). Changes in methylation state were defined using a HpaII/MspI ratio threshold of zero, where methylated loci and hypomethylated loci had ratio values less than zero and greater than zero, respectively. Ordered lists of differences were generated, the first relying purely on paired t-test computed p-values. Our alternative approach identified differential methylation, ranked according to the formula: 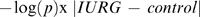 This method, similar to the modified T test [39], places more weight on the fold-change relative to the within group variability. However, unlike p-values based on the modified T test, computed values are not interpreted in these studies as a probabilistic quantity, and instead are used exclusively to rank and isolate important loci.

### Bisulphite MassArray Validation

Target regions were amplified by PCR using the primers and cycling conditions described in **Table S2**. Primers were selected with MethPrimer (http://www.urogene.org/methprimer/) using parameters as follows: 200–400 bp amplicon size, 56–60°C Tm, 24–30 bp length, and ≥1 CG in product. 50 µl PCR reactions were performed using the Roche FastStart High Fidelity Kit. In cases where products showed primer-dimer or other contaminants, bands of appropriate size were excised from 2% agarose gels, purified by Qiagen Gel Extraction Kit, and eluted with 1X Roche FastStart High Fidelity Reaction Buffer (+MgCl_2_). All PCR products (5 µl) were aliquotted onto 384-well microtiter plates and were treated with 2 µl of Shrimp Alkaline Phosphatase (SAP) mix for 20 minutes at 37°C to dephosphorylate unincorporated dNTPs. Microtiter plates were processed by the MassArray Matrix Liquid Handler. A 2 µl volume of each SAP-treated sample was then heat-inactivated at 85°C for 5 minutes and subsequently incubated for 3 hours at 37°C with 5 µl of Transcleave mix (T or C Cleavage Mix) for concurrent *in vitro* transcription and base-specific cleavage. Samples were transferred onto the spectroCHIP array by nanodispensation calibrated to ambient temperature and humidity, and analysis with the Sequenom MALDI-TOF MS Compact Unit following 4-point calibration with oligonuculeotides of different mass provided in the Sequenom kit. Matched peak data was exported using EpiTYPER software and analyzed for quality and single nucleotide polymorphisms according to analytical tools that we have recently developed [40].

### Ingenuity Pathway Analysis

The most significantly differentially methylated loci were mapped to RefSeq gene identifiers by chromosomal position within 10 kb upstream of the transcription start site or overlapping the gene body. The list of RefSeq identifiers was then uploaded to the Ingenuity Pathway Analysis program (Redwood City, CA), enabling exploration of ontology and molecular interaction networks. Each uploaded gene identifier was mapped to its corresponding gene object (focus genes) in the Ingenuity Pathways Knowledge Base. Core networks were constructed for both direct and indirect interactions using default parameters, and the focus genes with the highest connectivity to other focus genes were selected as seed elements for network generation. New focus genes with high specific connectivity (overlap between the initialized network and gene's immediate connections) were added to the growing network until the network reached a default size of 35 nodes. Non-focus genes (those that were not among our differentially methylated input list) that contained a maximum number of links to the growing network were also incorporated.

The ranking score for each network was then computed by a right-tailed Fisher's exact test as the negative log of the probability that the number of focus genes in the network is not due to random chance. Similarly, significances for functional enrichment of specific genes were also determined by the right-tailed Fisher's exact test, using all input genes as a reference set.

## Results

### Gestational and Neonatal Clinical Parameters in IUGR and Controls

Umbilical cord blood samples were collected from 10 well-matched, consenting individuals: IUGR (n = 5), and appropriate for gestational age controls (n = 5), matched for gestational age at delivery, gender, and ethnicity). We controlled for variable cellular composition of mixed leukocyte populations in the cord blood by purifying for hematopoietic stem (CD34+) cells, which served as a reference cell type for comparison in all subjects, thus reducing this potential source of variability which could have otherwise influenced our subsequent methylation assays [41]. We also note that while CD34+ cells are functionally heterogeneous themselves [42], purification reduces cell-specific variability in cytosine methylation that could otherwise mask (or artificially create) IUGR-related changes.

By design, IUGR weighed less and had a lower ponderal index than their matched controls, which were required to have normal percentiles for birth weight and ponderal index (**Table 1**). Both groups had similar gestational age at delivery and proportions of each sex and ethnic composition, and comparable maternal age, BMI, pregnancy weight gain, and 1-hour maternal plasma glucose screen values after a 50 g glucose load (**Table 1**). All of the women and their infants were healthy. None of the mothers had chronic hypertension, pre-eclampsia or gestational hypertension, pre-gestational or gestational diabetes, renal or autoimmune disease. The neonates had similar 1-minute and 5-minute Apgar scores in both groups (data not shown). One infant in the IUGR group had unilateral mild pyelectasis, which did not require treatment. There were no other neonatal complications.

**Table 1:** Neonatal and Maternal Characteristics for IUGR and Controls.

Neonatal and Maternal Characteristics	IUGR	Controls
	(n = 5)	(n = 5)
Gestational age, weeks (mean ±SD)	39.9±0.2	40.2±0.6
Birthweight, g (mean±SD)	2493±270	3240±276*
Ponderal index, g/cm^3^ (mean±SD)	2.3±0.08	2.81±0.1*
% Male	40	40
Maternal age, years (mean ±SD)	23.6±5.7	22.8±4.7
Pre-pregnancy BMI, kg/m^2^ (mean±SD)	24.5±3.2	25.1±7.5
Weight gain, pounds (mean±SD)	32±14	26±7
1-hour glucose screen, mg/dL (mean±SD)	93.8±25	74.3±26

* p<0.05 compared to matched cases.

### Distinct Patterns of DNA Methylation in IUGR and Control Subjects

To detect global patterns of epigenetic changes that could distinguish between IUGR and controls, we performed the HELP assay on the CD34+ cells. These genome-wide cytosine methylation profiles are available as a public resource through http://greallylab.aecom.yu.edu/~greally/humanIUGR/ and through the GEO repository (accession number GSE17727). We compared the full dataset of HELP data from IUGR and normal birthweight subjects using unsupervised clustering. We observed consistent patterns of methylation across all samples, without any apparent global changes in methylation status (*e.g.* the hypomethylation commonly seen in cancer [43]) between IUGR and control groups (**Figure S1**). Global Pearson correlation coefficients were calculated for the methylation patterns for each pair of samples, confirming a high degree of inter-sample consistency (R = 0.90−0.95).

**Figure 1:**
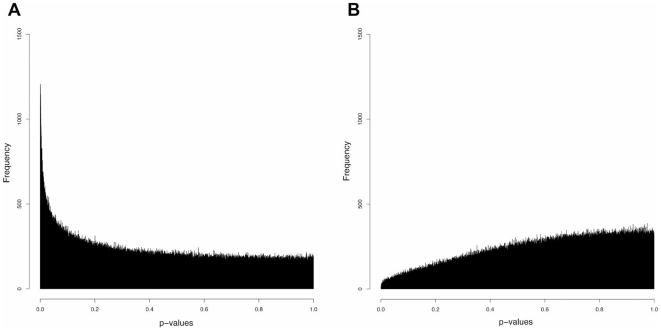
Supervised group comparisons reveal significant differences in HELP data results between IUGR and controls. Panel (A) shows a histogram distribution of p-values calculated from an unpaired T test of IUGR (n = 5) in one group and controls (n = 5) in another. The x axis represents p values, with lower values being the more significant, while the y axis shows the frequency of occurrence of different p values. The peak observed represents a subset of loci with low p values and thus significant differences between IUGR and control subjects. For comparison, panel (B) shows the results of a random distribution of subjects into two groups, mixing IUGR and controls, demonstrating the absence of a subset of loci with significant p values.

We tested to see whether cytosine methylation changes were present but undetected in global comparisons because they occur at only a subset of loci and/or to a limited degree at each locus. Paired T tests were applied to detect group differences for all individual loci represented on our arrays. This analysis yielded a subset of potentially informative loci with statistically significant differences in methylation. **Figure 1A** shows the distribution of p-values observed for IUGR compared to controls. **Figure 1B** shows the distribution of p-values when class labels are randomly permuted once across all loci and are representative of what we would see under the null distribution of no group effect. This illustrates how the observed p-values for the IUGR/control comparison are associated with a distribution consistent with lower than expected p-values for a small subset of loci, with 56 candidate loci (**Table 2**) having significant group differences (p<0.00001, chosen because no test statistics derived under any other permutation obtained a p-value below 0.000015), and an additional 646 loci with moderate group differences (p<0.0001).

**Table 2:** Characteristics of top 56 candidate loci identified by HELP.

Position	IUGR	Control	Difference	P	CpG	CGc	phC	Rep	Loc	Gene(s)	RefSeq
chr2:235526053-235526128	2.06	2.66	−0.61	0.4	YES	YES	YES		PRO	SH3BP4	NM_014521
chrX:66138356-66139206	−0.78	−1.29	0.51	0.8	no	no	no	RT			
chr15:45018938-45019873	−0.62	−1.11	0.48	0.9	no	no	YES				
chr6:106879977-106880120	2.56	3.34	−0.78	1.1	YES	YES	YES		PRO	ATG5	NM_004849
chr18:18624869-18626554	−0.72	−1.28	0.55	1.3	no	no	YES				
chr2:176841167-176842343	−0.16	−0.48	0.33	1.5	no	YES	no	LT	PRO	MTX2	NM_006554
chr6:113687585-113688546	−1.81	−2.61	0.80	1.5	no	no	YES				
chr11:77123865-77124724	−0.60	−1.33	0.73	1.8	YES	no	YES		GB	RSF1	NM_016578
chrX:70857596-70857847	0.51	1.28	−0.77	2.0	no	no	no				
chr19:2616301-2616955	−0.86	−1.18	0.32	2.2	no	no	no	RT	GB	GNG7	NM_052847
chr12:123573299-123573372	1.80	2.79	−1.00	2.2	no	no	no		PRO	NCOR2	NM_006312
chr4:102487593-102487694	1.59	2.55	−0.97	2.4	YES	YES	YES		PRO	PPP3CA	NM_000944
chr11:13612440-13613698	−1.01	−1.79	0.78	2.5	no	no	no				
chr20:39422191-39423123	−0.42	−0.94	0.52	2.7	no	no	YES		GB	LPIN3 EMILIN3	NM_022896 NM_052846
chrX:48943071-48943319	1.94	2.72	−0.78	3.0	YES	YES	YES		PRO	SYP	NM_003179
chr12:105961981-105963288	−1.44	−2.49	1.06	3.1	no	no	no	RT	PRO	CRY1	NM_004075
chr11:76172201-76172653	2.48	3.16	−0.68	3.2	YES	YES	YES		PRO	TSKU	NM_015516
chr8:318760-319070	−1.07	−0.60	−0.47	3.4	no	no	no				
chr8:25575968-25577680	−1.53	−2.41	0.88	3.4	no	no	YES				
chrX:99552722-99552801	3.09	3.62	−0.52	3.7	YES	YES	YES		PRO	PCDH19	NM_020766
chr22:45949018-45950112	−1.23	−1.87	0.65	3.7	no	YES	no				
chr11:99814353-99814807	−0.48	0.36	−0.83	3.8	no	no	no				
chr15:91226882-91227081	2.40	3.34	−0.93	3.8	no	YES	YES				
chr3:9809841-9809915	1.49	2.24	−0.74	3.8	YES	YES	YES		PRO2	TADA3L ARPC4	NM_006354 NM_005718
chr12:1774845-1774939	1.72	2.45	−0.73	4.2	no	no	no		GB	CACNA2D4	NM_172364
chr15:88120371-88120587	1.28	2.08	−0.79	4.4	YES	YES	YES		PRO	MESP2	NM_001039958
chr9:78132928-78133704	−0.91	−1.56	0.65	5.2	no	no	YES				
chr9:33740402-33740551	1.61	2.41	−0.80	5.2	YES	YES	no		PRO	PRSS3	NM_007343
chr16:4746813-4747596	−1.50	−1.85	0.35	5.2	no	no	no	RT	GB	ZNF500	NM_021646
chr3:129693359-129693420	1.66	2.31	−0.65	5.2	YES	YES	no		PRO	GATA2	NM_032638
chr2:206657793-206657892	2.47	3.43	−0.96	5.4	no	YES	YES		PRO	INO80D	NM_017759
chr6:21543836-21545402	−1.61	−2.10	0.49	5.6	no	no	no	RT			
chr22:16347210-16348259	−1.26	−1.62	0.36	5.9	no	no	no	RT	GB	CECR2	NM_031413
chr8:94370466-94372334	−1.26	−2.09	0.84	6.1	no	no	no				
chr20:22581679-22582432	−1.09	−1.76	0.67	6.1	no	no	YES				
chr21:25927844-25929694	−1.13	−1.77	0.64	6.3	no	no	YES	RT/LT			
chr12:105841213-105843079	−1.42	−1.71	0.29	6.4	no	no	no	RT			
chrX:39752669-39752723	1.04	1.99	−0.95	6.5	YES	YES	YES				
chr16:87294955-87295022	1.03	1.63	−0.60	6.7	no	YES	no		GB	RNF166	NM_178841
chr22:17514809-17516325	2.52	3.26	−0.74	7.0	YES	YES	no		PRO	GSC2	NM_005315
chr15:78244066-78244975	−1.04	−1.49	0.45	7.1	no	no	no		GB	FAH	NM_000137
chr10:101825755-101826964	−1.72	−2.11	0.39	7.2	no	no	YES		PRO	CPN1	NM_001308
chr16:29563652-29563719	2.38	3.22	−0.83	7.3	no	no	no	RT			
chr5:132564040-132565611	−1.89	−2.55	0.66	7.5	no	no	YES		PRO	FSTL4	NM_015082
chr18:40597598-40598729	−2.17	−2.86	0.69	7.6	no	no	YES		GB	SETBP1	NM_015559
chr17:17019265-17021036	−1.34	−1.94	0.60	7.9	no	no	YES		GB	MPRIP	NM_015134
chr4:101159747-101160275	−1.72	−2.38	0.66	8.0	no	no	no	RT			
chr11:120258272-120258456	2.13	2.91	−0.78	8.1	no	no	YES		PRO	GRIK4	NM_014619
chr9:19033554-19034579	−0.19	−0.84	0.65	8.5	no	no	no	RT			
chr8:59144552-59145204	−1.10	−1.61	0.52	8.6	no	no	YES		GB	FAM110B	NM_147189
chr6:41861921-41862019	0.99	1.56	−0.57	8.7	YES	YES	YES		GB	PRICKLE4	NM_013397
chr15:61088671-61090467	−0.44	−1.03	0.59	8.8	no	no	no				
chr10:71811777-71811840	1.86	2.76	−0.90	8.8	YES	YES	no		PRO	LRRC20	NM_018205
chr12:109990645-109990725	1.32	2.32	−1.00	9.1	no	no	YES		PRO	CUX2	NM_015267
chr3:124611357-124611643	3.63	4.23	−0.61	9.4	no	no	YES		PRO	ADCY5	NM_183357
chr10:92344854-92345619	−1.70	−2.71	1.01	9.7	no	no	no				

All positions correspond to coordinates in the human genome, hg18 March 2006 UCSC Genome Browser; IUGR and Control data given as group averages of log_2_(HpaII/MspI); difference is IUGR minus Control; P-values are all x10^−6^; **CpG**, overlap with CpG islands; **CGc** overlap CG clusters; **phC** overlap with mammalian or vertebrate phastCons conserved elements; **Rep** overlap with repetitive elements (**RT** for retrotransposable elements including LINEs and SINEs, **LT** for long terminal repeats); **Loc** overlap with promoters (**PRO**), bidirectional promoters (**PRO^2^**), or gene bodies (**GB**) of RefSeq genes; Gene names and corresponding RefSeq identifiers are also shown.

For comparison, p-value distributions calculated for grouping by gender and ethnicity are shown in **Figures S2A and S2B**, respectively. Gender comparison shows ∼2,500 significant loci (p<0.00001 threshold), an expected outcome due to the vast majority of these discriminatory loci (99.4%) being located on the X and Y chromosomes. We found no evidence for ethnicity (Latino versus non-Latino) influencing cytosine methylation (**Figure S2B**).

When we looked in detail at the 56 differentially-methylated loci, we found that the magnitude of DNA methylation changes observed in IUGR compared to controls is markedly less than many tissue-specific differences in methylation that we have previously observed ([44]; and data not shown). Furthermore, we analyzed methylation status at four known imprinted regions on chromosome 11, some of which have been shown to harbor small changes in methylation in subjects with IUGR (the *IGF2* differentially-methylated region (DMR), the *H19* DMR and promoter region, and the *KCNQ1* DMR) [25], [45] but found no detectable differences in the IUGR group compared to controls in this particular cell type (data not shown).

### Validation Studies on Selected Candidate Loci

The next component of our two-stage experimental design was to validate the HELP data with single-locus, nucleotide-resolution quantitative studies, for which we used bisulphite MassArray [34]. To choose the loci for validation, we assessed the candidacy of the top 56 loci in terms of related biological function, reasoning that epigenetic dysregulation of the small degrees we were observing would have to affect multiple components of a biological pathway to cause functionally-significant changes. We used an Ingenuity Pathway Analysis (IPA) for 33 of the top 56 candidate loci, chosen because of physical proximity to 35 RefSeq-annotated genes (22 promoters and 13 gene bodies). While the remaining 23/56 loci in intergenic regions could not be linked to genes using these proximity criteria, almost half of these loci occur at CG clusters [46] or phastCons conserved DNA elements [47], both characteristics having potential relevance as *cis*-regulatory sites [48] and therefore may have unrecognized roles regulating transcription.

**Figure 2:**
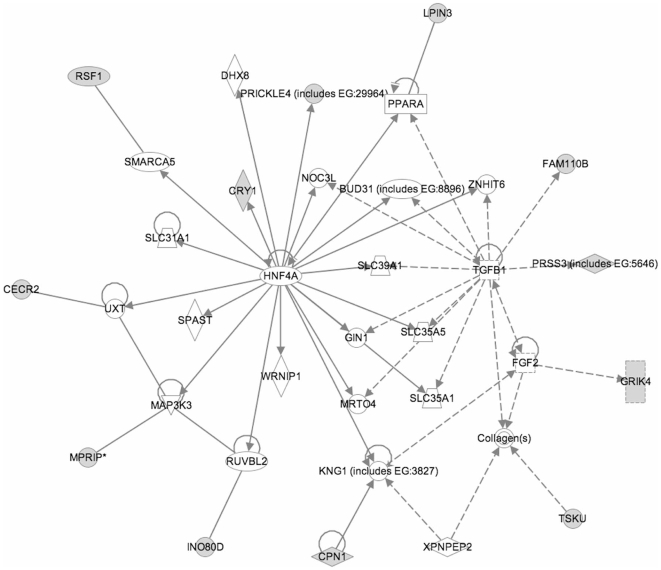
A second molecular interaction network suggested by Ingenuity Pathway Analysis (IPA), with HNF4A as a central node, consists of 12 genes among the top 56 differentially methylated loci. RefSeq IDs for 33 of the top 56 sites that mapped to genes were uploaded onto the “Core Analysis” tool of IPA. The second-highest scoring molecular interaction network was constructed by 35 nodes, 12 of which were located on the input list (shaded nodes), and is associated with the cell cycle, cellular function and maintenance, and connective tissue development and function. The nodal relationships are indicated by solid lines (direct interaction) and dashed lines (indirect interactions), with or without filled arrows indicating functional interaction or merely physical association, respectively. Additionally, filled arrows that are preceded by a terminal bar indicate inhibition as well as functional interaction. The shape of each node indicates the class of molecule: horizontal ovals are transcription factors, squares are growth factors, vertical rectangles are ion channels while horizontal rectangles are nuclear receptors, inverted triangles are kinases, vertical diamonds are enzymes while horizontal diamonds are peptidases, trapezoids are transporters, and circles correspond to “other” molecules. In alphabetical order, this network consists of BUD31, CECR1, Collagen(s), CPN1, CRY1, DHX8, FAM110B, FGF2, GIN1, GRIK4, HNF4A, INO80D, KNG1, LPIN3, MAP3K3, MPRIP, MRTO4, NOC3L, PPARA, PRICKLE4, PRSS3, RSF1, RUVBL2, SLC31A1, SLC35A1, SLC35A5, SLC39A1, SMARCA5, SPAST, TGFB1, TSKU, UXT, WRNIP1, XPNPEP2, and ZNHIT6.

Of the two highest-scoring molecular interaction networks generated from IPA analysis, one was associated with cell signaling, nucleic acid metabolism, and molecular transport (**Figure S3**), while the other network contained 12 out of 35 input nodes and was functionally associated with the cell cycle, cellular maintenance, and connective tissue development (**Figure 2**). Moreover, this network was centered primarily on the hepatic nuclear factor 4α (*HNF4A*), a transcription factor that has been strongly implicated in an early-onset form of type II diabetes, maturity onset diabetes of the young [49]. Although HNF4A did not meet our strict threshold for significance in the primary analysis, it was ranked first in our secondary analysis using a test statistic that was more heavily weighted towards the mean shift between groups (see **Methods**).

An additional IPA that was applied specifically to candidate loci overlapping RefSeq gene promoters revealed a third molecular interaction network including 17 of 23 input nodes. This network is functionally associated with cancer, cellular development, cellular growth and proliferation (**Figure S4**), and is centered on a number of transcription factors and growth hormones (e.g. *NCOR2*, *GH1*, *TGFβ1*, *HNF4A*), and *TP53* which shows altered DNA methylation in renal tissue of IUGR rats in adult life [26].

We therefore proceeded to bisulphite MassArray experiments [34], initially testing the technical performance of the HELP assays by choosing four loci, two each representing constitutively hypomethylated and constitutively methylated sites identified by the HELP assay. **Figure S5** demonstrates the agreement between methylation values determined independently for these loci by HELP and MassArray (with a between-assay correlation in this case of R = −0.96353).

**Figure 3:**
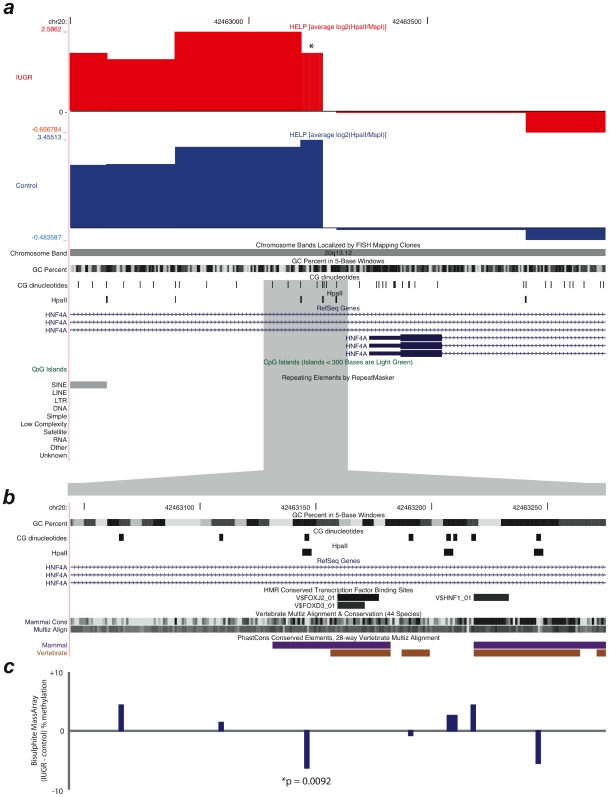
Differential methylation at the *HNF4A* locus proximal promoter region. HELP data are shown in (a) as normalized, centered log_2_(HpaII/MspI) ratios. A locus with a change in methylation is marked with an asterisk. A more detailed view of this region is shown in (b), showing conservation of DNA sequences at this alternative promoter of *HNF4A*. In (c) the degree of difference in cytosine methylation as measured by bisulphite MassArray is shown with the locus changing to a significant degree shown with its associated p value. This CG dinucleotide is within one of the HpaII sites of the informative gragment in (a) and is located immediately beside the conserved transcription factor binding sites shown in (b). These images were derived from the UCSC Genome Browser [60].

The technical validation results confirmed that the HELP data were accurate and allowed us to proceed to the analysis of the specific loci at which dysregulation of cytosine methylation was suspected. We focused on the *HNF4A* locus, where HELP data showed evidence for changes in cytosine methylation at an internal promoter of this complex gene (**Figure 3**). Furthermore, the identified locus represents highly conserved sequence and contains multiple known transcription factor binding sites for HNF1α and β, SP1, HNF6, and GATA6 [50]. Differences in DNA methylation at the *HNF4A* promoter were confirmed by MassArray, with the informative HpaII site showing hypermethylation in IUGR compared to controls (65.7% and 59.6% methylation, respectively; p<0.01), consistent with the HELP data (log_2_(HpaII/MspI) = 1.89±0.42 and 3.46±0.60, respectively; p = 0.00006). This increased methylation level is of a magnitude comparable with that observed in samples from the Dutch famine cohort [25]. Functionally, the acquisition of cytosine methylation at this promoter may reduce *HNF4A* expression in IUGR offspring, consistent with the inherited loss-of-function mutations in HNF4A that lead to an autosomal dominant form of maturity onset diabetes of the young (MODY) [49].

### Effect Size and Power Computations

One of our goals was to use our data *post hoc* to define how best to design this kind of study to test the dysregulation of cytosine methylation in a human disease. We therefore calculated an estimation of effect size for the top 1,000 loci with differences in methylation between IUGR and controls. Similar to the distribution of methylation differences noted for the top 56 loci, the 1,000 most informative loci had a mean difference of 0.60 with a standard deviation of 0.20 log_2_ units. The difference in average methylation between control and IUGR at all loci measured tended to be subtle (between 0.20 and 1.00) and less likely to represent broad methylation state changes (going from a completely methylated state to a hypomethylated state and *vice versa*). We simulated informative genes with this information as a guide for true group differences in this population (**Table 3**).

**Table 3:** Estimation of power for different study sample sizes.

Sample size per group	Power at alpha = 0.05	Power at alpha = 1e−5	Power at alpha = 1e−10
5	0.74	0.003	<0.0001
10	0.98	0.11	0.0002
15	1.00	0.45	0.00
25	1.00	0.94	0.12
35	1.00	1.00	0.57
50	1.00	1.00	0.97
100	1.00	1.00	1.00

cases: N(0.60, 0.32).

controls: N(0.00, 0.32).

## Discussion

We present preliminary evidence for a unifying new hypothesis in which epigenetic modification induced in early development may contribute to an increased susceptibility to age-related diseases by prematurely advancing the normal aging process. Using a genome-wide, high-resolution cytosine methylation assay (testing ∼1.32 million loci throughout the genome), we avoid the necessity for any *a priori* assumptions about candidate loci and reveal potential new candidates in mediating the fetal origin of adult disease. We identified dysregulation of DNA methylation in purified populations of cord blood-derived CD34+ stem cells from IUGR neonates compared with matched controls, and find that these changes occur throughout the genome, although they are of a more modest degree and relatively limited extent compared with the epigenomic dysregulation observed in conditions such as cancer. As our study was based on a limited number of subjects, such subtle differences in cytosine methylation challenge the ability of genome-wide approaches to identify genuinely significant loci even at standard significance levels (0.05–0.001) which do not reflect multiple testing. In large-scale studies, when thousands to millions of loci are measured, the threshold to declare any locus significant is typically based on a more stringent level of significance (*e.g.* Bonferroni correction or similar) in order to minimize the possibility of false positives, therefore we have displayed the power at two such thresholds. Based on these simulations, a more expansive approach, with sample size of at least 25 subjects per group is recommended for studies of methylation changes in conditions where such subtle effects are expected.

Nonetheless, we found that the IUGR subjects were distinctive for having a number of consistent differences in methylation near genes involved in processes critical for stem cell function, including cell cycle and cellular maintenance. Quantitative bisulphite validation studies confirmed our ability to discriminate differences in methylation in these samples, and the biological coherence of results in terms of functional pathway relatedness is suggestive of underlying changes in epigenetic regulation as a response to IUGR.

A locus that emerged consistently in this pathway analysis was the *HNF4A* gene, already implicated in T2DM [49], but not previously demonstrated to undergo epigenetic dysregulation as a response to IUGR. Best known for its implications as a monogenic, autosomal dominant form of maturity onset diabetes of the young (MODY) [49], HNF4A is involved in development and function of both the liver and the pancreas [51] and actively coordinates gene expression of many important metabolic pathways in both tissues [52], [53], [54]. We find differences in DNA methylation targeted to only one of the *HNF4A* promoters, supporting a model of isoform variation of the gene being related to susceptibility to T2DM, a major age-related disease.

Other loci identified in this study were found to be related functionally to *HNF4A* and also include *ATG5* and *TADA3L*, which may have roles in mediating susceptibility to later disease. *ATG5* is an essential component of autophagy that, when depleted, renders cells more susceptible to starvation and starvation-induced cell death [55]. We found that *ATG5* is relatively methylated in IUGR at a CpG island-containing site just downstream of the transcription start site, potentially reducing expression in IUGR compared with controls, in parallel with increased sensitivity to starvation-induced cell death. Transcriptional adaptor 3 (*TADA3L*) is associated with and is required for full p53 activity, causing growth arrest, senescence, and p53-mediated apoptosis [56]. *TADA3L* isoforms are highly expressed in CD34+ stem cells [57], but we find that *TADA3L* is relatively methylated in IUGR at a CpG island-containing bidirectional promoter, potentially downregulating its expression and altering CD34+ stem cell population dynamics.

However, the modest level of differences in methylation that we and others have observed [25] raises an important question: what is the biological significance of changes in methylation on the order of ∼6%? We note that such a change must represent a difference in a proportion of cells and/or alleles undergoing methylation within the broader population of CD34+ cells. Because CD34+ stem cells are multipotent progenitors, the presence of an epigenetically dysregulated subpopulation may go on to mediate susceptibility to chronic disease, with potentially greater effects over time should this subpopulation expand. Alternatively, this stem cell population may serve to define loci susceptible to constitutive “epimutations” [58] that are likely to exist in descendent cell types or unrelated lineages (*e.g.* liver or pancreatic progenitors) where they may have the chance to induce functional changes in critical cell types or tissues. Adding further information about epigenetic and transcriptional regulators other than cytosine methylation plus transcriptional profiling studies will be very valuable in gaining a greater understanding of the epigenetic dysregulation and its functional consequences in IUGR.

We hypothesize that the changes we observe by focused studies of hematopoietic (CD34+) stem cells are representative of the influence of the intrauterine environment on epigenetic regulation and independent cellular programs throughout the developing fetus. While all cells are believed to accumulate epimutations over time [59], periods of rapid cell division (*e.g.* during fetal development) represent the most vulnerable windows for cellular injury and potential dysregulation of the epigenome, with associated decreases in cellular fitness, function, and, of particular note for multipotent stem cells, replicative capacity. We therefore propose that adverse intrauterine conditions are more likely to contribute to replicative senescence and early exhaustion of regenerative pools of stem cell precursors throughout the body, increasing susceptibility to and speeding onset of age-related diseases like T2DM.

Although the exact mechanism remains unclear, the results of this study are indicative of epigenetic dysregulation associated with intrauterine growth restriction. Moreover, these findings suggest that epigenetic changes serve as a steward of cellular memory of aberrant intrauterine environments, and that site-specific changes in DNA methylation may mediate the increased susceptibility to age-related diseases observed later in life.

### Accession Numbers

GEO database, accession number GSE17727.

## Supporting Information

Figure S1 Global correlation using the entire dataset demonstrates a high degree of epigenetic correlation among all samples, with no apparent global difference between IUGR and controls. A tree-pair plot was generated using a visualization tool that we have developed as part of an R package (Thompson, 2008). Pairwise correlations, calculated from >1.32 million independent loci, are shown in the upper right portion of the figure, where R values indicate the Pearson correlation for each pair of samples (labeled along the diagonal) and blue dotplots show a visual representation of the similarity between samples. Thin red lines within each of these sub-panels correspond to a lowess-fit of the pairwise data. In the lower left portion of the figure, a tree determined by Ward's minimum variance clustering shows an alternative unsupervised clustering approach. Branching order is shown in solid lines, colored by group. The diagonal dotted lines are numbered and indicate the Euclidean distance scale. The dotted red line indicates the Euclidean distance cutoff used to separate the individual groups of samples. Thompson, R.F., Reimers, M., Khulan, B., Gissot, M., Richmond, T.A., Chen, Q., Zheng, X., Kim, K. and Greally, J.M. (2008) An analytical pipeline for genomic representations used for cytosine methylation studies, Bioinformatics, 24, 1161–1167.
(0.70 MB TIF)

Figure S2 Supervised group comparisons reveal differences in methylation based on gender but not ethnicity. Panel (A) shows a histogram distribution of p-values calculated from an unpaired T test of males (n = 4) in one group and females (n = 6) in another. Along the x-axis, leftmost data represent low p-values (i.e. highly significant differences in methylation between groups), while data towards the right represent high p-values and, thus, loci that are uniform across groups. P-value frequency is shown along the y-axis, with larger values indicating increasing numbers of differentially methylated loci corresponding to the indicated p-value level. Note that the y-axis in this panel is adjusted to a different scale in order to account for the higher frequency of highly significant p-values. Panel (B) shows an analogous histogram with data obtained from samples grouped by ethnicity (Latin, n = 7, compared to non-Latin, n = 3).
(0.04 MB TIF)

Figure S3 The top-scoring molecular interaction network suggested by Ingenuity Pathway Analysis (IPA) consists of 13 genes among the top 56 differentially methylated loci. RefSeq IDs for 33 of the top 56 sites that mapped to genes were uploaded onto the “Core Analysis” tool of IPA. The top-scoring molecular interaction network was constructed by 35 nodes, 13 of which were located on the input list (shaded nodes), and is associated with cell signaling, nucleic acid metabolism, and molecular transport. The nodal relationships are indicated by solid lines (direct interaction) and dashed lines (indirect interactions), with or without filled arrows indicating functional interaction or merely physical association, respectively. The shape of each node indicates the class of molecule: horizontal ovals are transcription factors, squares are cytokines, vertical rectangles are G-protein coupled receptors, triangles are phosphatases, diamonds are enzymes, trapezoids are transporters, small ovals are chemicals, and circles correspond to “other” molecules. In alphabetical order, this network consists of ADCY, ADCY5, ADCY9, ARPC4, ATG5, β-estradiol, CAP2, CRYM, CUX2, EMILIN3, FSH, GALR1, GALR3, GH1, GJA1, GNAI2, GNAL, GNG7, GSTM3, HTR1F, LPAR4, MAMLD1, 5-methoxytryptamine, MllRN181C, NCOR2, noladin ether, PALM, PCDH19, PPP3CA, RXFP4, SH3BP4, SYP, TADA3L, and TP53.
(0.32 MB TIF)

Figure S4 A molecular interaction network suggested by Ingenuity Pathway Analysis (IPA) “Core Analysis”, with HNF4A as a central node among other transcription and growth factors. This network, associated with cancer, cellular development, cellular growth and proliferation, consists of 17 of 23 input nodes, each corresponding to the promoter of a RefSeq gene showing differential methylation by HELP. Nodes are shaded in green for relative hypermethylation in IUGR compared to controls, while red-shaded nodes are hypomethylated in IUGR. The nodal relationships are indicated by solid lines (direct interaction) and dashed lines (indirect interactions), with or without filled arrows indicating functional interaction or physical association, respectively. The shape of each node indicates the class of molecule: horizontal ovals are transcription factors, squares are growth factors, vertical rectangles are ion channels, triangles are phosphatases, diamonds are enzymes, trapezoids are transporters, and circles correspond to “other” molecules with concentric circles indicated complexes. In alphabetical order, this network consists of ADCY5, ARPC4, ATG5, β-estradiol, BUD31, C11ORF10, C9ORF5, Ca2+, CDKN2A, CHCHD8, CPA2, CPN1, CRY1, CTNNBL1, CUX2, FSH, GATA2, GH1, GINS3, GRIK4, HNF4A, INO80D, NCOR2, PPP3CA, PRSS3, RUVBL2, SH3BP4, SLC35A1, SLC35A5, SPP1, SYP, TADA3L, TGFB1, TP53, and TSKU.
(0.46 MB TIF)

Figure S5 Technical validation studies using MassArray confirm HELP data. Four loci were identified, two each representing constitutively hypo- and hyper-methylated sites. The hypomethylated sites were located at chr12:63986847-63987489 and chr20:45414330-45414885, with hypermethylated sites at chr4:188493650-188494271 and chr7:69549255-69549831 (hg18, human genome, March 2006, UCSC Genome Browser). HELP data as log2(HpaII/MspI) ratios are shown along the x-axis, with methylation towards the left and hypomethylation towards the right. MassArray data for the same loci are plotted along the y-axis, from 0% (hypomethylated) to 100% (methylated).
(0.03 MB TIF)

Table S1 Neonatal and Maternal Characteristics for IUGR and Controls
(0.03 MB DOC)

Table S2 Characteristics of top 56 candidate loci identified by HELP. All positions correspond to coordinates in the human genome, hg18 March 2006 UCSC Genome Browser; IUGR and Control data given as group averages of log_2_(HpaII/MspI); difference is IUGR minus Control; P-values are all x10^−6^; CpG, overlap with CpG islands; CGc overlap CG clusters; phC overlap with mammalian or vertebrate phastCons conserved elements; Rep overlap with repetitive elements (RT for retrotransposable elements including LINEs and SINEs, LT for long terminal repeats); Loc overlap with promoters (PRO), bidirectional promoters (PRO^2^), or gene bodies (GB) of RefSeq genes; Gene names and corresponding RefSeq identifiers are also shown.
(0.04 MB DOC)
