# Supplementary figures and images for: Cytosine Methylation Dysregulation in Neonates Following Intrauterine Growth Restriction
Source: PLoS One. 2010 Jan 26;5(1):e8887. doi: 10.1371/journal.pone.0008887 (PMC2811176; doi:10.1371/journal.pone.0008887)

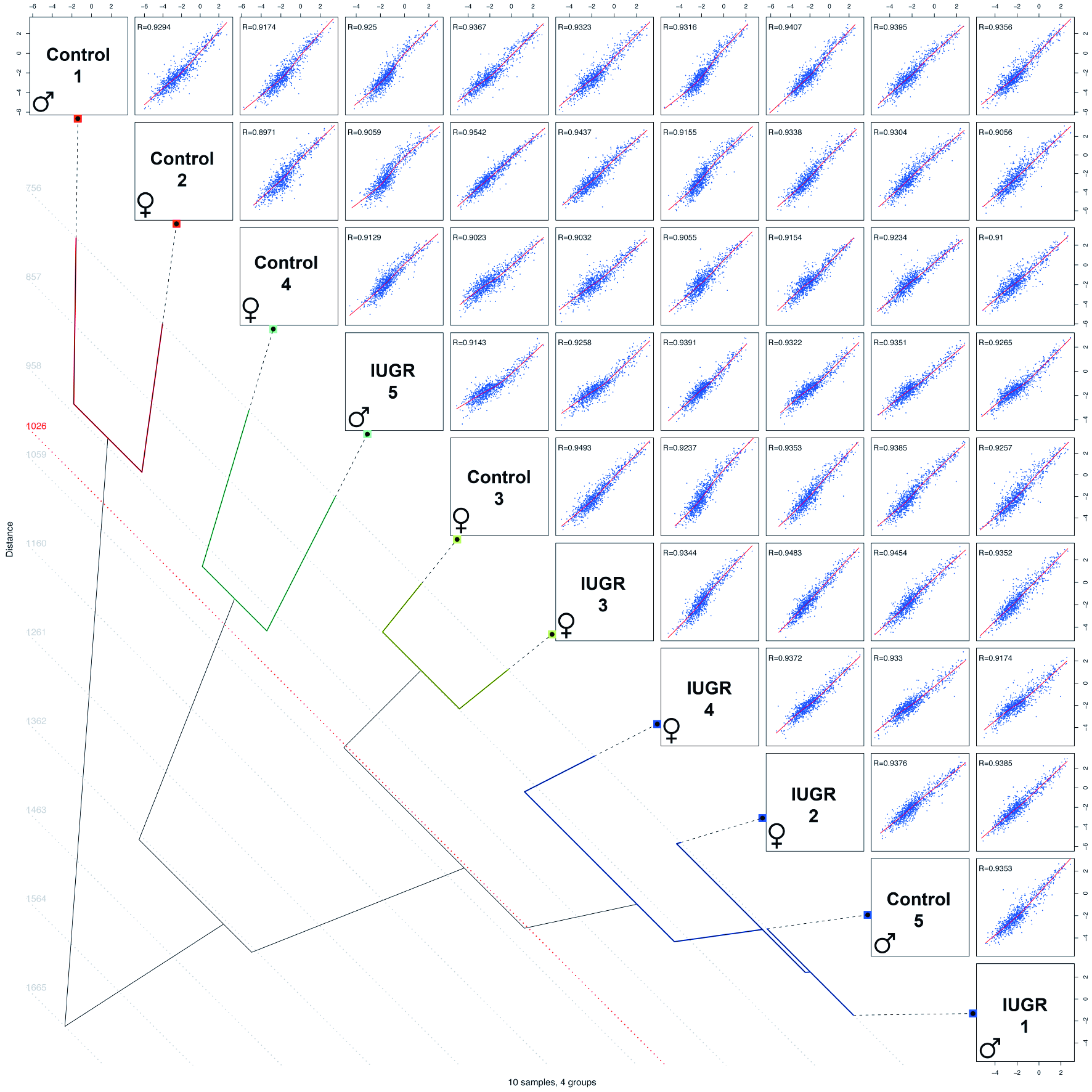

Supplement: Figure S1 — Global correlation using the entire dataset demonstrates a high degree of epigenetic correlation among all samples, with no apparent global difference between IUGR and controls. A tree-pair plot was generated using a visualization tool that we have developed as part of an R package (Thompson, 2008). Pairwise correlations, calculated from >1.32 million independent loci, are shown in the upper right portion of the figure, where R values indicate the Pearson correlation for each pair of samples (labeled along the diagonal) and blue dotplots show a visual representation of the similarity between samples. Thin red lines within each of these sub-panels correspond to a lowess-fit of the pairwise data. In the lower left portion of the figure, a tree determined by Ward's minimum variance clustering shows an alternative unsupervised clustering approach. Branching order is shown in solid lines, colored by group. The diagonal dotted lines are numbered and indicate the Euclidean distance scale. The dotted red line indicates the Euclidean distance cutoff used to separate the individual groups of samples. Thompson, R.F., Reimers, M., Khulan, B., Gissot, M., Richmond, T.A., Chen, Q., Zheng, X., Kim, K. and Greally, J.M. (2008) An analytical pipeline for genomic representations used for cytosine methylation studies, Bioinformatics, 24, 1161–1167. (0.70 MB TIF) [file pone.0008887.s001.tif]

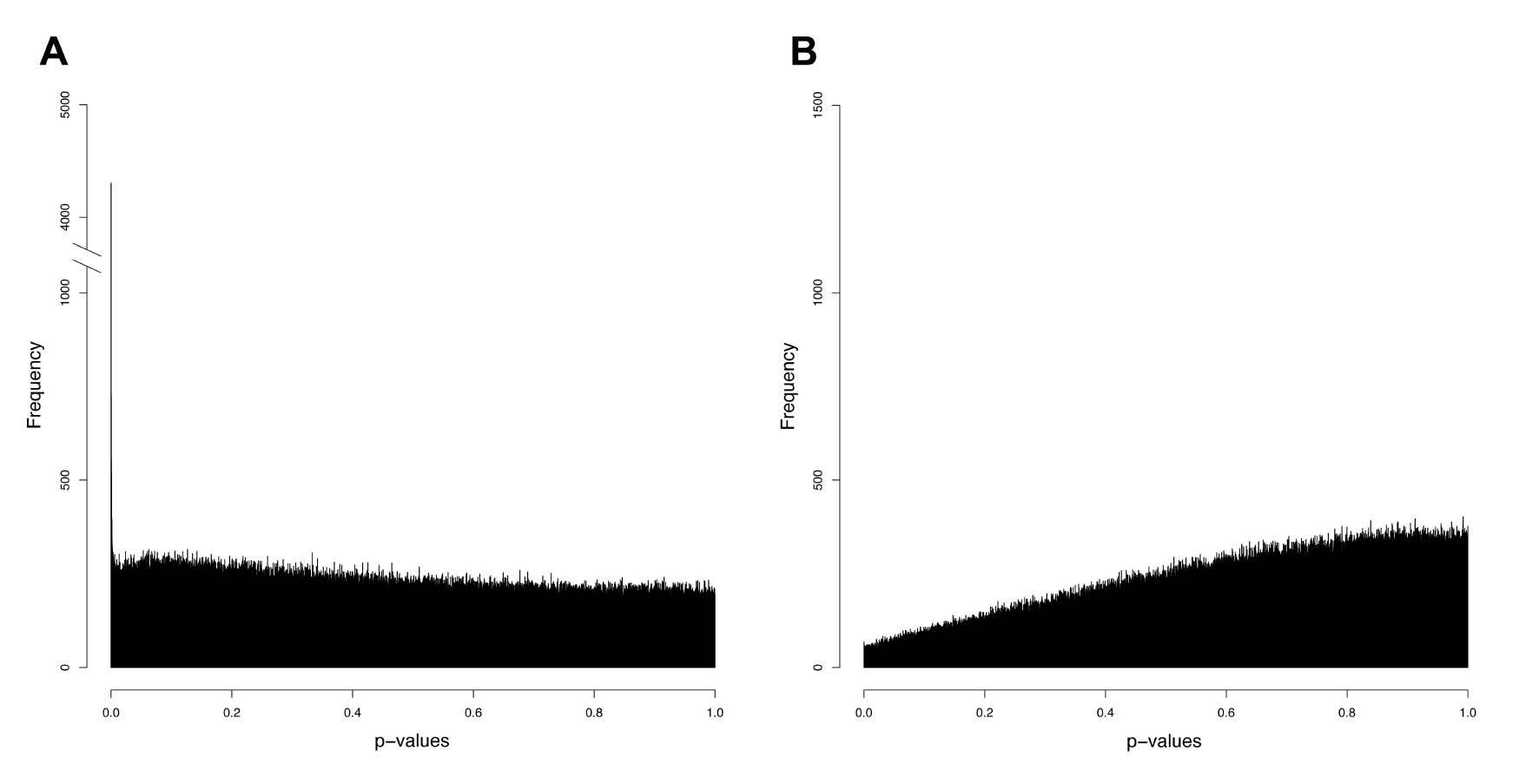

Supplement: Figure S2 — Supervised group comparisons reveal differences in methylation based on gender but not ethnicity. Panel (A) shows a histogram distribution of p-values calculated from an unpaired T test of males (n = 4) in one group and females (n = 6) in another. Along the x-axis, leftmost data represent low p-values (i.e. highly significant differences in methylation between groups), while data towards the right represent high p-values and, thus, loci that are uniform across groups. P-value frequency is shown along the y-axis, with larger values indicating increasing numbers of differentially methylated loci corresponding to the indicated p-value level. Note that the y-axis in this panel is adjusted to a different scale in order to account for the higher frequency of highly significant p-values. Panel (B) shows an analogous histogram with data obtained from samples grouped by ethnicity (Latin, n = 7, compared to non-Latin, n = 3). (0.04 MB TIF) [file pone.0008887.s002.tif]

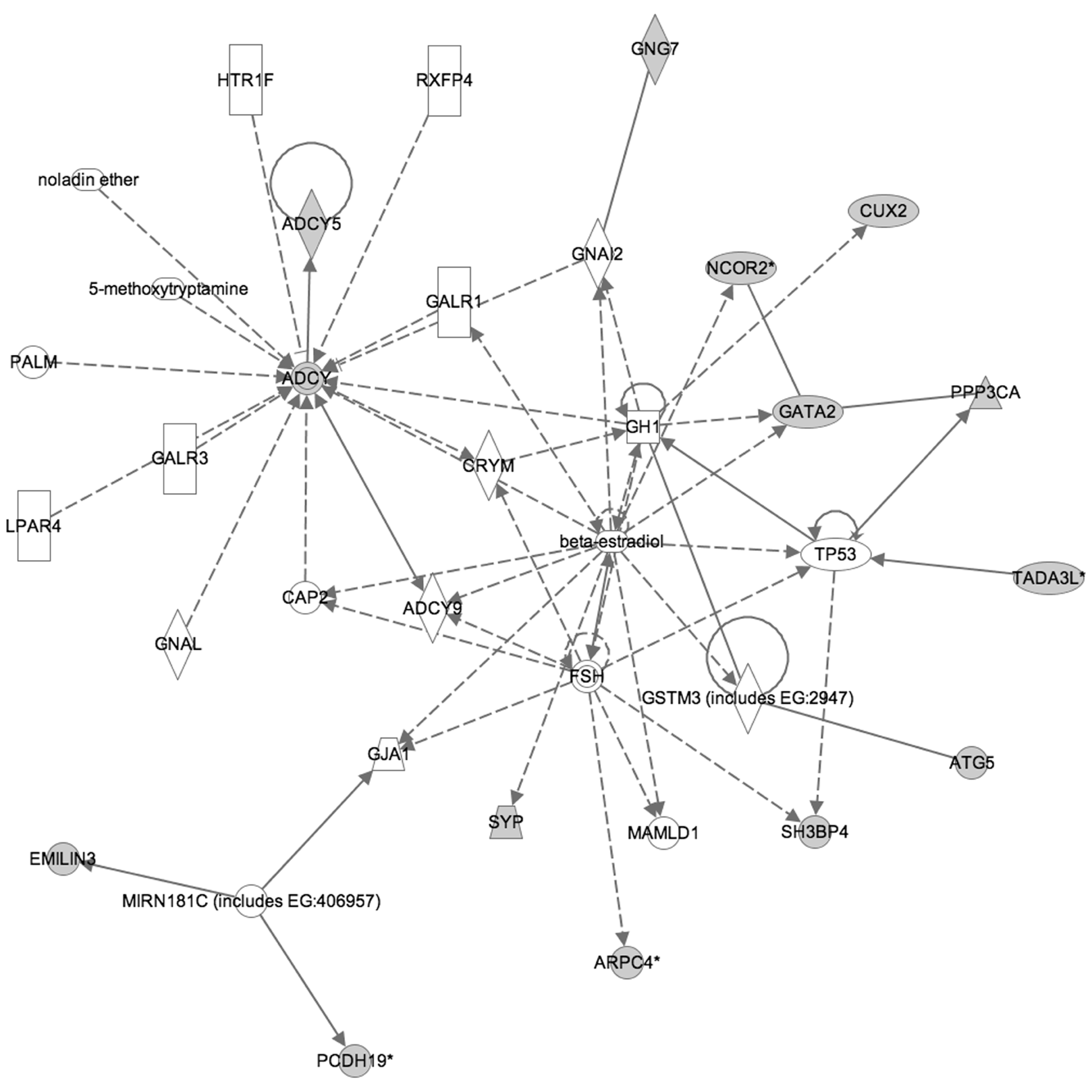

Supplement: Figure S3 — The top-scoring molecular interaction network suggested by Ingenuity Pathway Analysis (IPA) consists of 13 genes among the top 56 differentially methylated loci. RefSeq IDs for 33 of the top 56 sites that mapped to genes were uploaded onto the “Core Analysis” tool of IPA. The top-scoring molecular interaction network was constructed by 35 nodes, 13 of which were located on the input list (shaded nodes), and is associated with cell signaling, nucleic acid metabolism, and molecular transport. The nodal relationships are indicated by solid lines (direct interaction) and dashed lines (indirect interactions), with or without filled arrows indicating functional interaction or merely physical association, respectively. The shape of each node indicates the class of molecule: horizontal ovals are transcription factors, squares are cytokines, vertical rectangles are G-protein coupled receptors, triangles are phosphatases, diamonds are enzymes, trapezoids are transporters, small ovals are chemicals, and circles correspond to “other” molecules. In alphabetical order, this network consists of ADCY, ADCY5, ADCY9, ARPC4, ATG5, β-estradiol, CAP2, CRYM, CUX2, EMILIN3, FSH, GALR1, GALR3, GH1, GJA1, GNAI2, GNAL, GNG7, GSTM3, HTR1F, LPAR4, MAMLD1, 5-methoxytryptamine, MllRN181C, NCOR2, noladin ether, PALM, PCDH19, PPP3CA, RXFP4, SH3BP4, SYP, TADA3L, and TP53. (0.32 MB TIF) [file pone.0008887.s003.tif]

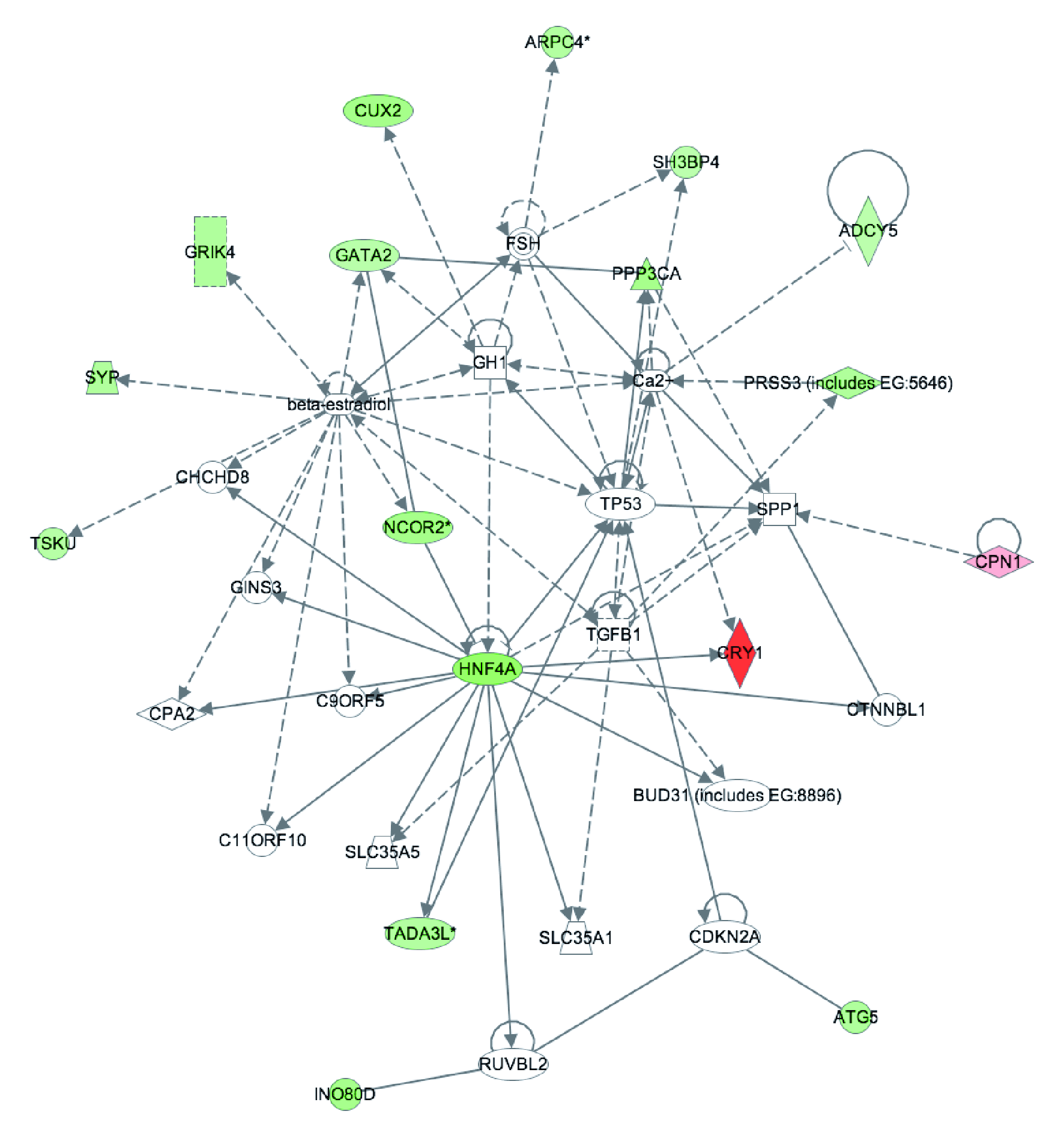

Supplement: Figure S4 — A molecular interaction network suggested by Ingenuity Pathway Analysis (IPA) “Core Analysis”, with HNF4A as a central node among other transcription and growth factors. This network, associated with cancer, cellular development, cellular growth and proliferation, consists of 17 of 23 input nodes, each corresponding to the promoter of a RefSeq gene showing differential methylation by HELP. Nodes are shaded in green for relative hypermethylation in IUGR compared to controls, while red-shaded nodes are hypomethylated in IUGR. The nodal relationships are indicated by solid lines (direct interaction) and dashed lines (indirect interactions), with or without filled arrows indicating functional interaction or physical association, respectively. The shape of each node indicates the class of molecule: horizontal ovals are transcription factors, squares are growth factors, vertical rectangles are ion channels, triangles are phosphatases, diamonds are enzymes, trapezoids are transporters, and circles correspond to “other” molecules with concentric circles indicated complexes. In alphabetical order, this network consists of ADCY5, ARPC4, ATG5, β-estradiol, BUD31, C11ORF10, C9ORF5, Ca2+, CDKN2A, CHCHD8, CPA2, CPN1, CRY1, CTNNBL1, CUX2, FSH, GATA2, GH1, GINS3, GRIK4, HNF4A, INO80D, NCOR2, PPP3CA, PRSS3, RUVBL2, SH3BP4, SLC35A1, SLC35A5, SPP1, SYP, TADA3L, TGFB1, TP53, and TSKU. (0.46 MB TIF) [file pone.0008887.s004.tif]

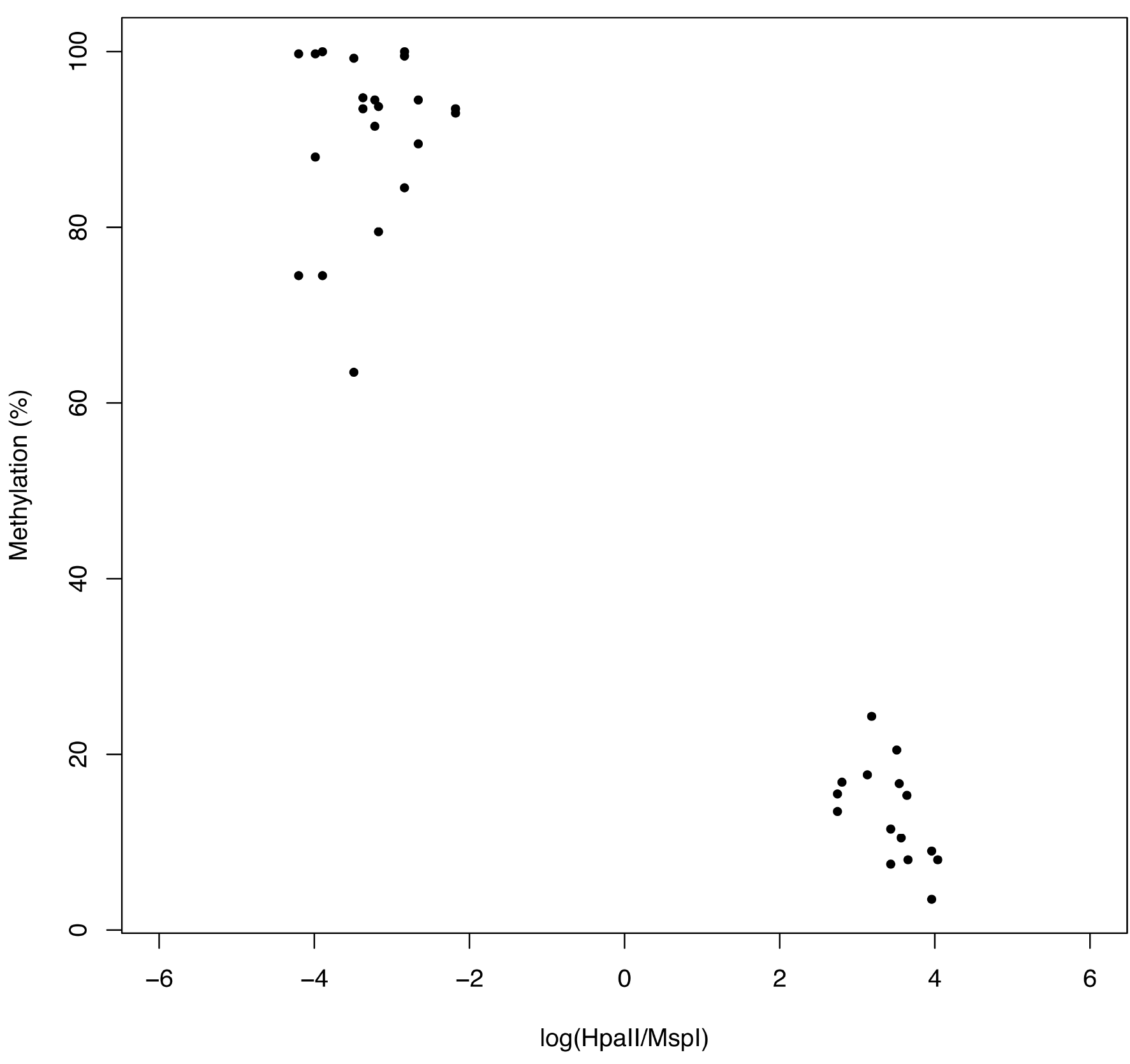

Supplement: Figure S5 — Technical validation studies using MassArray confirm HELP data. Four loci were identified, two each representing constitutively hypo- and hyper-methylated sites. The hypomethylated sites were located at chr12:63986847-63987489 and chr20:45414330-45414885, with hypermethylated sites at chr4:188493650-188494271 and chr7:69549255-69549831 (hg18, human genome, March 2006, UCSC Genome Browser). HELP data as log2(HpaII/MspI) ratios are shown along the x-axis, with methylation towards the left and hypomethylation towards the right. MassArray data for the same loci are plotted along the y-axis, from 0% (hypomethylated) to 100% (methylated). (0.03 MB TIF) [file pone.0008887.s005.tif]
