# Supplementary material for: Cytosine Methylation Dysregulation in Neonates Following Intrauterine Growth Restriction
Source: PLoS One. 2010 Jan 26;5(1):e8887. doi: 10.1371/journal.pone.0008887 (PMC2811176; doi:10.1371/journal.pone.0008887)
Supplement: Table S1 — Neonatal and Maternal Characteristics for IUGR and Controls (0.03 MB DOC) [file pone.0008887.s006.doc]

| **Table S1** | | |  |  |  |  |
| --- | --- | --- | --- | --- | --- | --- |
| Sample characteristics and pairwise groupings for analysis by T test | | | | | | |
| **CONTROL** | | | | **IUGR** | | |
| **Gender** | **Gestational Age** | **Ethnicity** | | **Gender** | **Age** | **Ethnicity** |
| Male | 40 | Latin | | Male | 39.9 | Latin |
| Male | 40 | Caucasian | | Male | 39.7 | Caucasian |
| Female | 40 | Latin | | Female | 40.1 | Black |
| Female | 39.4 | Latin | | Female | 39.7 | Latin |
| Female | 41 | Latin | | Female | 40 | Latin |
