# Supplementary material for: Cytosine Methylation Dysregulation in Neonates Following Intrauterine Growth Restriction
Source: PLoS One. 2010 Jan 26;5(1):e8887. doi: 10.1371/journal.pone.0008887 (PMC2811176; doi:10.1371/journal.pone.0008887)
Supplement: Table S2 — Characteristics of top 56 candidate loci identified by HELP. All positions correspond to coordinates in the human genome, hg18 March 2006 UCSC Genome Browser; IUGR and Control data given as group averages of log2(HpaII/MspI); difference is IUGR minus Control; P-values are all x10−6; CpG, overlap with CpG islands; CGc overlap CG clusters; phC overlap with mammalian or vertebrate phastCons conserved elements; Rep overlap with repetitive elements (RT for retrotransposable elements including LINEs and SINEs, LT for long terminal repeats); Loc overlap with promoters (PRO), bidirectional promoters (PRO2), or gene bodies (GB) of RefSeq genes; Gene names and corresponding RefSeq identifiers are also shown. (0.04 MB DOC) [file pone.0008887.s007.doc]

| **Table S2.**  Primer pairs and cycling conditions for amplicons used in this study. | | | | |  |
| --- | --- | --- | --- | --- | --- |
|  | **AMPLICON** | **PRIMERS** | | | **Tm (°C)** |
|  |  | Forward^*^ | Reverse^T7^ | |  |
|  | chr4:188493538-188493834 | AATATTGTTAGGATATTGGGTAGTTTATAG | AAAACTAATAAAAAAATAATTCACCTCTAC | | 57 |
|  | chr4:188494066-188494394 | GAGGATGTTTTATATTGATGAGTTGG | AAACCAATATTCTCAAAAACCTACAAC | | 59 |
|  | chr7:69549072-69549443 | TATTTTGATAGAGGTTTGGGTTTATTAG | AAATCACATTATCCATAAAAAACAAATTAC | | 58 |
|  | chr7:69549694-69550115 | TTAAGGGTTTTGATTTTTTGTTTAGAGG | AAAACTCTATCAAACTCAATCCAACTAC | | 57 |
|  | chr12:63986773-63987118 | AGTTTTGGTTATTTGGGTAGAAATATAG | ACCCTTCTCTATAAAAAATAAAACACAC | | 56 |
|  | chr12:63987260-63987548 | TTAATGGTTTTTTTAGGTTGTGTATTGT | TATCTTATTCATTTCCTAACCACTTCC | | 59 |
|  | chr20:45414163-45414450 | GAGTTTTTAATATGAGGTTTTGTGATG | CAACCCTTTAACTTAACTTTTCAAATAC | | 58 |
|  | chr20:45414660-45414981 | GAGTTTGAGAATTAATAATGGGTTTTG | CTTACAAACAAACAAACAAACAATAAC | | 59 |
|  | chr20:42462991-42463280 | AGTATTAGTTAGAATGTTTGATTTGGG | AACCTAACCTCTATAAAAAAATAAAAACTC | | 57 |
| Tm, annealing temperature in degrees centrigrade used for PCR amplification; amplicon coordinates according to human genome version hg18 Mar. 2006, UCSC Genome Browser. | | | | | |
| ^*^ Forward primer sequences listed all contain a 10bp tag at their 5’ ends (AGGAAGAGAG) | | | |  | |
| ^T7^ Reverse primer sequences listed all contain a 31bp tag at their 5’ ends (CAGTAATACGACTCACTATAGGGAGAAGGCT) | | | |  | |
